# Effects of Socioeconomic Status on Colorectal Cancer Incidence and Clinical Outcome Differences Among Asian American Populations: A Systematic Review

**DOI:** 10.7759/cureus.83806

**Published:** 2025-05-09

**Authors:** Amy Sakazaki, Austin Lui, Melody Wang, Katherine Ngo, Maria Theresa Lugue, Himani Aligireddy, Maria Nguyen, Christianne L Castro, Kenneth Park, Shin Murakami

**Affiliations:** 1 College of Osteopathic Medicine, Touro University California, Vallejo, USA

**Keywords:** asian subgroups, colorectal cancer, genetic background, socioeconomic factors, systematic review and meta-analysis

## Abstract

Colorectal cancer (CRC) is a leading cause of cancer death among Asian Americans and Pacific Islanders (API) in the United States. Race, ethnicity, and socioeconomic status (SES) are known to impact outcomes of CRC, but the relationship is unclear in the context of the Asian American cohort and its diverse subgroups. This systematic review aims to gain insight into the relationship between CRC incidence and clinical outcomes in the Asian American community. A systematic literature search was conducted per the Preferred Reporting Items for Systematic Reviews and Meta-analyses (PRISMA) protocol using PubMed, Scopus, Excerpta Medica database (EMBASE), Cochrane, Cumulative Index to Nursing and Allied Health Literature (CINAHL), and Web of Science databases, accessed on August 13, 2023. Of the 2225 studies identified, a total of 14 studies were included in the analysis. Four studies concluded that there was no association or variable response subgroup-specific to SES measurements in CRC incidence in the Asian American population. However, there was evidence that the incidence of CRC varies among Asian American subgroups, using varying measures of SES. Seven of the eight studies that measured mortality or survival as the primary outcome found decreased mortality and increased survival in the API population despite changes in SES. Out of the six studies that measured incidence, four studies found no association with SES. A study found that Chinese Americans had a significant decrease in the CRC incidence and mortality across all SES categories. Japanese Americans experienced a significant decrease in the lowest SES category, while Koreans and Filipinos experienced a significant increase in both the lowest and highest SES categories. Therefore, grouping various Asian American ethnicities as a single monolithic "Asian" category is misleading. Although the incidence of CRC was thought to be low and decreasing, this review identified various subgroup-specific trends among 24 different Asian American subsets. For example, there was a decrease in CRC rates in two ethnic groups and an increase in the other two ethnic groups. The potential causes of these varying CRC incidence rates are likely multifactorial and may include inadequate screening rates, lack of CRC education, and cultural barriers. Further studies are needed to understand these mechanisms. This review recommends a more detailed classification of the API ethnic population but not as a single monolithic entity as Asian. It also emphasizes preventative CRC screening within the API communities due to lower rates of CRC screening among them.

## Introduction and background

Colorectal cancer (CRC) is the third most common cancer and the third leading cause of cancer deaths in the United States [1]. It is projected that in 2023, an estimated 153,020 individuals will be diagnosed with CRC, and approximately 52,550 will die from the disease [1]. Established risk factors include advanced age, genetics, and socioeconomic status (SES), such as income, education level, insurance, and geographical location [2]. Socioeconomic and cultural factors could also affect potential modifiable risk factors, including smoking, a diet high in processed or red meats and low in fruits and vegetables, high alcohol consumption, physical inactivity, and excess body weight [3]. Clinical outcomes of CRC have been improving nationally, likely due to increases in rates of CRC screening [4]. Screening rates also likely represent the ability and accessibility of care.

CRC is the second most common cancer among Asian Americans, while it is third overall in the US population; one in 22 men (4.6%) and one in 24 women (4.2%) will face a diagnosis in their lifetime [5]. After a rise from 1975 to 1985, incidence rates have decreased significantly, which is driven by effective cancer prevention strategies and the widespread adoption of early screening measures [5]. Asian Americans represent 6.2% of the US population and are the fastest-growing racial or ethnic group. According to 2019 data, the Asian American population has increased by 81% in the past two decades [6]. The most prominent subgroups within this population include Chinese, Indian, Filipino, Vietnamese, Korean, and Japanese; however, over 20 detailed subgroups are listed on the US census [7]. This population embodies a diverse population with an expansive range of languages, histories, cultures, and SES. Therefore, each group has its own set of health behaviors, genetic and cultural risks, and clinical outcomes. There is a varying degree of acculturation to having a more Western lifestyle and diet, which leads to an increased body weight and decreased physical activity.

Despite the large diversity among the Asian ethnic groups, the medical literature tends to treat them as a single monolithic entity, often together with Asian Americans and Pacific Islanders (API) [5,8]. This leads to the misleading assumption that all Asian Americans have a comparable health status, despite the inherent diversity among them. Although higher incidence was associated with lower SES in non-Hispanics, the CRC rates are higher for Hispanic and Asian women living in higher SES areas, who also experience elevated rates of CRC [9]. For example, it has been reported that Asian Americans, as a monolithic group, have comparable or favorable prognoses compared to other racial or ethnic groups [10-14]. However, when examined as separate ethnic or racial identities, there is considerable variation in incidence trends and outcomes [15]. Mortality rates from CRC also varied, with Native Hawaiians and Southeast Asians having the greatest risk of mortality from CRC; however, Chinese, Japanese, and Indians/Pakistanis had a lower risk, according to the California Department of Public Health and registries, which participated in the National Cancer Institute's Surveillance, Epidemiology, and End Results (SEER) program [16,17].

The widening of SES disparity in the last several decades has correlated with mortality from all cancers, including CRC, and extends to cardiovascular disease as well [18]. Few studies have examined the effects of SES on clinical outcomes in the context of heterogeneous Asian American ethnicities, justified by including over 20 subgroups and 15 keywords relating to SES variables. With the Asian American population projected to reach 43 million by the year 2050, the factors that affect incidence rates and outcomes of CRC in the Asian American population must be well-characterized to inform preventative measures. Therefore, this systematic review seeks to delineate whether SES factors affect clinical outcomes of CRC in the Asian American population. We hope to elucidate which ethnic groups within the Asian American population and what SES factors will impact the clinical outcomes of CRC to focus efforts on cultural and structural implementations for the greatest impact.

This article was previously posted to the medRxiv preprint server on February 21, 2025.

## Review

Methods

Review and Search Strategy

This systematic review was conducted in accordance with the Preferred Reporting Items for Systematic Reviews and Meta-analyses (PRISMA) 2020 criteria for transparency and used Covidence systematic review software (Veritas Health Innovation, Melbourne, Australia) [19,20] for optimal efficiency and reviewer collaboration when screening. We systematically searched PubMed, Scopus, EMBASE, Cochrane, CINAHL, and Web of Science (last accessed on August 13, 2023). The keywords included Asian Americans, AAPI, Pacific Islander, as well as all Asian subgroups combined with "American" e.g., "Vietnamese American", AND colorectal cancer, colorectal neoplasm, colorectal tumor, colon cancer, rectal cancer, AND social determinants of health, healthcare disparities, socioeconomic status, socioeconomic factors neighborhood SES, poverty, social class, income, insurance, occupation, geographic location, literacy, inequality, education, employment, home environment, ethnic enclave, clinical outcomes, treatment outcomes, incidence, mortality, morbidity, vital statistics, progression-free survival, prognosis, intraoperative complications, intraoperative complication, surgical complication, patient outcomes assessment, outcome, critical care outcome, patient care, length of stay, discharge, hospitalization, long term care, palliative care, terminal care, hospice care, rehabilitation, activities of daily living. An example of the complete search strategy can be found in the appendix.

Asian American Subgroups

Asian American subgroups that were included were Asian Indian, Bangladeshi, Bhutanese, Burmese, Cambodian, Chinese, Filipino, Hmong, Indian, Indonesian, Japanese, Korean, Laotian, Malaysian, Mongolian, Nepalese, Pakistani, Singaporean Americans, South Asian, Sri Lankan, Thai, Tibetan, Taiwanese, and Vietnamese Americans.

Inclusion Criteria

The criteria for inclusion included the following: peer-reviewed, written in the English language, studies that analyzed incidence rate and/or clinical outcomes (treatment outcomes, incidence, mortality, complications, progression-free survival, etc.) among patients diagnosed with CRC, studies that included Asian American patients as a racial/ethnic group, and studies that discussed indicators of SES (demographics, socioeconomic status, income, education, geographical location, etc.) as a variable on clinical outcomes. We used p < 0.05 as a threshold for SES variables. There were no restrictions on the date of publication. The accepted study types included retrospective, prospective, cross-sectional, randomized, nonrandomized, or crossover controlled studies, case series, and case reports. The bias of the studies was assessed in the Results section.

Exclusion Criteria

Letters, commentaries, conference abstracts not meeting the inclusion criteria, reviews, systematic reviews, meta-analyses, and studies without full text available were excluded. We excluded any texts written in a non-English language. We considered conference abstracts; however, none met the inclusion criteria.

Study Selection

We used Covidence software [20] to further screen the titles and abstracts of the remaining studies. Seven independent reviewers assessed the studies to ensure they met the defined inclusion and exclusion criteria. Discrepancies were discussed and resolved by a panel of two reviewers. Data extraction was completed manually by two reviewers.

Bias Assessment

The risk of bias was assessed using the ROBINS-E tool. Studies were assessed according to ROBINS-E [21] criteria by one reviewer in seven domains: confounding, measurement of exposure, selection of participants, post-exposure intervention, missing data, measurement of the outcome, selection of reported results, and overall bias judgment. The Robvis tool was used to visualize the risk of bias assessment [22].

Results

**Figure 1:**
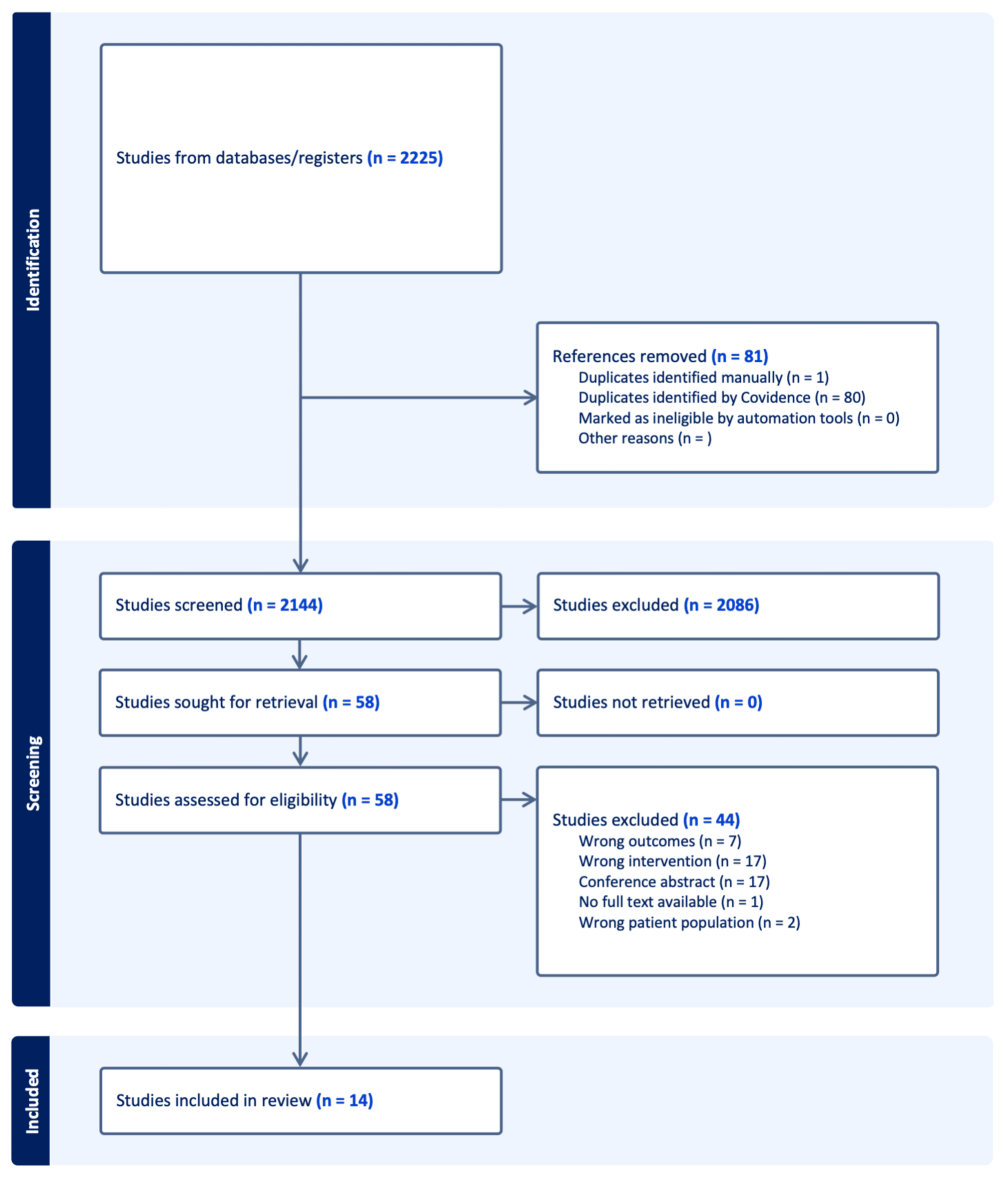
PRISMA flow diagram depicting the study selection process PRISMA: Preferred Reporting Items for Systematic Reviews and Meta-analyses

We conducted a comprehensive literature search, as described in the Methods section, which identified a total of 2,225 studies. After excluding 80 duplicate entries, we used Covidence software to further screen the titles and abstracts of the remaining studies. Seven independent reviewers assessed the studies to ensure they met the defined inclusion and exclusion criteria. Initially, 38 studies were selected for full-text review by two independent reviewers. Subsequently, several studies were excluded from consideration: seven studies did not measure the incidence of CRC or relevant clinical outcomes, 17 studies failed to assess SES indicators, and 17 conference abstracts were disregarded. Additionally, one study was excluded due to the unavailability of the full text, and two studies were removed because they did not involve populations within the United States. In total, 14 studies were selected for further data retrieval, as shown in Figure 1.

The 14 articles identified (Table 1) were all retrospective studies that investigated several SES variables, including urban vs. rural (in two studies), poverty levels (in two studies), SES quintiles or composite SES score (in seven studies), ethnic enclaves (in two studies), and insurance type (in four studies). The outcomes measured in the studies were the incidence rate or rate ratio (in six studies), mortality rate (in five studies), survival rate (in three studies), and laparoscopic surgery (in one study). Additionally, four of the 14 articles analyzed at least one specific Asian American subgroup.

**Table 1:** Study characteristics * p < 0.05 ** p < 0.01 SES: socioeconomic status; SEER: National Program of Cancer Registries, National Cancer Institute's Surveillance, Epidemiology, and End Results

Study	Years Studied	Database	SES Variable	Outcome	Simplified Findings
Alnasser et al. 2013 [23]	2009	2009 Healthcare Cost and Utilization Project: Nationwide Inpatient Sample	Insurance type	Laparoscopic surgery	No significant association
Chu et al. 2007 [24]	1990–2000	National Center for Health Statistics, US Census	Poverty level	Mortality rate, rate ratio disparity measure	Mortality rates are down-trending, with low mortality disparity rates in all poverty levels
Coughlin et al. 2006 [25]	1998–2001	SEER	Urban vs. rural residence	Age-adjusted incidence rate	Increase in CRC incidence in metropolitan areas in comparison with rural residence*
Ellis et al. 2018 [26]	2000–2013	California Cancer Registry	SES composition, surgery, insurance composition, insurance type	Cancer-specific mortality	Lower mortality when compared to non-Hispanic Whites*
Enewold et al. 2014 [27]	1990–2007	SEER	Poverty level	Mortality rate	Mortality rates are down-trending, with relatively low mortality disparity rates in all poverty levels
Giddings et al. 2012 [28]	1988–2007	California Cancer Registry	Composite SES score	Age-adjusted incidence per 100,000	In every SES category, Chinese Americans experienced a decrease in incidence.** In the lowest SES, Japanese Americans experienced a decrease in incidence.* In the lowest SES, Filipino Americans experienced an increase in incidence.* In the lowest and highest SES, Korean Americans experienced an increase in incidence**
Kcomt and Gorey 2020 [29]	1995–2000	California Cancer Registry	Enclave residence Insurance type Marital Status	Mortality	In the less insured, improved mortality outcomes with enclave residence and married status*
Kish et al. 2014 [30]	2002–2008	SEER	SES quintiles	5-year cause-specific survival	Significantly higher survival in the highest SES quintile*
Krieger et al. 1999 [31]	1988–1992	Medical chart review, San Francisco Bay Area	Composite SES score	Incidence rate ratio	No significant association
Ladabaum et al. 2014 [32]	1994–2004	SEER	SES quintiles enclave residence	Age-adjusted incidence rate	No significant association
Lewis-Thames et al. 2022 [33]	1975–2011	SEER	Urban vs. rural residence	5-year survival rate	Significant increase of urban CRC survival in the years 1975–1986 and 1991–2011**
Pulte et al. 2017 [34]	2007–2013	SEER	Insurance type	Age-standardized survival	Difference in survival advantage between Medicaid and uninsured/other insurance was decreased in comparison to non-Hispanic Whites
Steinbrecher et al. 2012 [35]	1998–2002 and 1999–2001	California Cancer Registry, US Census	SES quintiles	Incidence rate, incident rate ratio, mortality rate ratio	No significant association
Yin et al. 2010 [8]	1998–2002	California Cancer Registry, US Census	Composite SES score	Age-adjusted incidence rate	No association seen in API men; API women in the highest SES experienced an increased incidence

Risk of Bias

**Figure 2:**
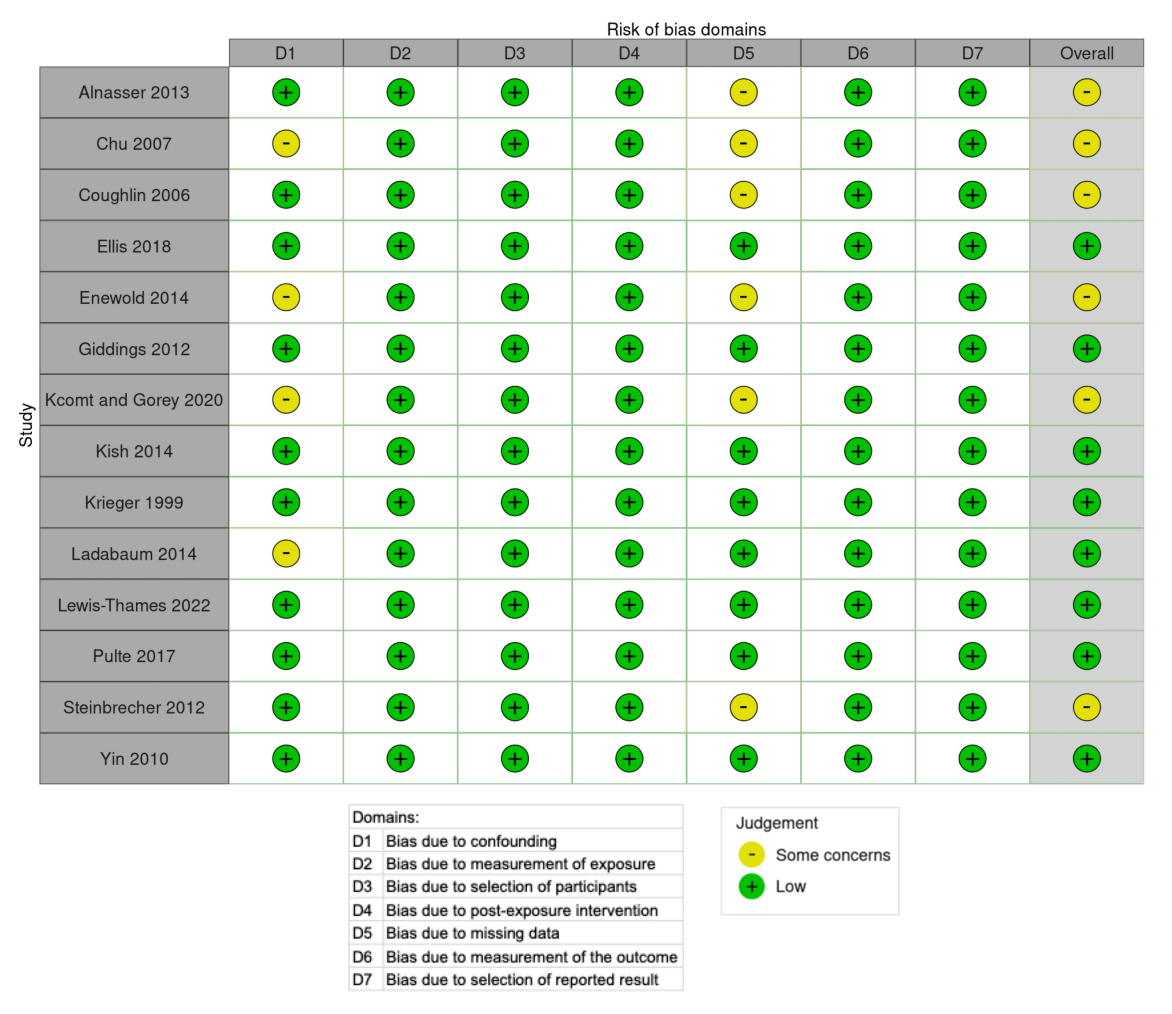
Risk of bias

The overall quality of evidence was "low risk" in 10 studies and "some concerns" in six studies. The studies were placed in the "some concerns" category due to potential confounding and missing data. None of the studies were deemed high risk or very high risk of bias (see Figure 2 for a visual representation).

Incidence

Six studies measured incidence as an outcome. One study found a significant increase (p < 0.05) in incidence in metropolitan areas (4396 cases) compared to rural areas in the API population (61 cases) [25]. When investigating incidence trends among Asian American subgroups, Giddings et al. (2012) found that, when measuring age-adjusted incidence, Chinese Americans experienced a significant decrease (p < 0.01) in every SES category; Japanese Americans experienced a significant decrease (p < 0.05) in the lowest SES; and Korean Americans and Filipino Americans experienced a significant increase (p < 0.01) in incidence both in the lowest and in the highest SES categories [28]. However, the study did not control for confounders, including lifestyle, family history, or other medical conditions, despite controlling for age at diagnosis and stratifying by sex. The other four studies found no clear association solely with SES measurements. Instead, they identified a combination of factors influencing SES, including the stage of disease at diagnosis, tumor site and progression, cultural shifts toward an American lifestyle and diet, poverty, and racial/ethnic disparities [24,26,27,35,36].

Mortality and Survival

Four out of the five studies indicated that mortality rates are lower in the API group compared to non-Hispanic Whites, despite changes in SES factors [24,26,27,35]. One study revealed that marital status and the presence of an ethnic enclave improved mortality, and Chinese Americans benefited more from enclaves than other ethnicities [29]. Similarly, studies found that, despite some SES-related differences [30], the API group had an increased survival rate overall. This was attributed to factors such as insurance type and access to healthcare screenings and treatments, alongside differing racial and ethnic disparities [30,33,34].

Discussion

**Figure 3:**
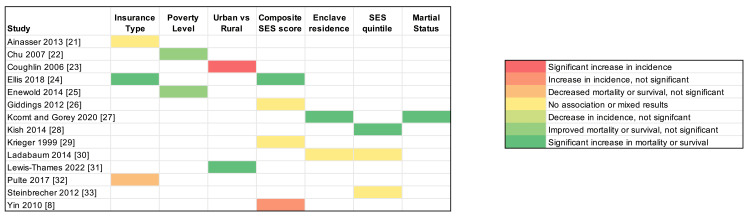
Heat map reflecting our study results SES: socioeconomic status

To our knowledge, this is among the first systematic reviews to assess the impact of SES on CRC outcomes, specifically within the Asian American population. Existing literature reports that overall, incidence and mortality rates associated with CRC in the API group are lower than those of non-Hispanic Whites [37]. Our findings align with the prevailing epidemiological consensus, indicating that Asian Americans exhibit improved CRC outcomes relative to other racial and ethnic groups. Nonetheless, this review also revealed significant heterogeneity in CRC outcomes when analyzing the population as a consolidated entity, as illustrated in Figure 3. Notably, there is emerging evidence suggesting that the incidence of CRC is on the rise in certain subgroups of Asian Americans, contrasting the national trends [32,36].

This review underscores that the designation of a singular monolithic "Asian" category is misleading in epidemiological assessments of CRC. When analyzed as distinct ethnic or racial identities, there is notable heterogeneity in incidence trends and outcomes within Asian populations [15]. Specifically, while the prevalence of CRC has increased among the API population, national trends show a decline, likely due to higher education levels and increased economic earnings [36]. A 1998 report highlighted that among Asian American subgroups, the highest incidence rates were observed in Japanese men (64.1 per 100,000) and Alaska Natives (79.7 per 100,000) [38]. Furthermore, a 2013 investigation illuminated increasing incidence rates among Korean and Native Hawaiian populations [39]. Notably, a population-based study revealed that Laotian, Samoan, and Vietnamese men were significantly less likely to receive diagnoses at earlier stages of CRC [40]. This disparity emphasizes the necessity for disaggregated data to depict the epidemiology of CRC across diverse Asian subpopulations accurately.

Secondly, mortality rates from CRC also varied among ethnic subgroups, with Native Hawaiians and Southeast Asians having the greatest risk of mortality from CRC, but Chinese, Japanese, and Indians/Pakistanis had a lower risk [16,17]. Japanese Americans in Hawaii also were reported to have improved survival rates in the long term [41]. Additionally, foreign-born Asian Americans are more likely to have increased mortality from CRC [42]. However, Chinese males and foreign-born Filipinos had lower CRC incidence compared to foreign-born Japanese, who had a higher incidence [30,40]. This difference likely arises from variations in lifestyle and healthcare access resulting from migration [32,43]. These studies highlight the heterogeneity in CRC statistics among Asian American groups. Some groups do demonstrate more favorable survival outcomes, but other groups are more at risk, a statistic that is overseen if all Asian Americans were treated monolithically [16]. Additionally, despite national improvement in screening that has contributed to an overall decrease in mortality, screening rates among Asian American groups have been inadequate [44,45]. This may be attributed to various factors, including a lack of awareness, the absence of physician recommendations, fear of a cancer diagnosis, time constraints, limited access to healthcare, lack of health insurance, limited English proficiency, low health literacy, lower socioeconomic status, low levels of acculturation, and other non-financial barriers [44,45].

Finally, the widening of SES disparity in the last several decades has correlated with mortality from all cancers and extends to cardiovascular disease as well [18]. It has been demonstrated that low SES, including poverty, lack of education, lack of social support, and social isolation, are associated with poorer survival in CRC [18,46]. Low SES also correlates to a lack of health insurance and access to care, which is a barrier to receiving appropriate screening, leading to later-stage disease at diagnosis and higher mortality [18]. However, SES and its relationship to disparities among race and ethnicity are complex, and studies have shown a variable association with outcomes [30,31]. Further, studies suggest that the extent to which SES factors, such as private health insurance or marital status, improve cancer survival can differ among race/ethnicity [11,47]. A more detailed ethnic classification of the API population is crucial for improving clinical outcomes. For example, if distinct genetic backgrounds are identified within each ethnicity, it could enable a more precise assessment and targeted treatment of CRC based on molecular mechanisms [48,49].

Importantly, despite a high incidence of CRC in specific Asian American subgroups, such as Chinese and Filipino, the API have a lower rate of CRC screening compared to White and African Americans. However, it has been reported that only 50% of Asian individuals have up-to-date colonoscopies compared to 61% of White and African Americans [1,44,50]. Several cultural factors contribute to this disparity, including a lack of CRC knowledge, less acculturation, cancer-related fatalism, poor English language proficiency, embarrassment about screenings, and limited social support, to name a few [51-55]. Previous studies have revealed the importance of culturally and socially appropriate counseling and guidance, as targeted efforts to respect diverse cultural beliefs and practices have been shown to decrease the barriers to screening [52,54]. For example, focus group studies among Korean Americans showed that recommendations from doctors and access to Korean physicians positively influenced decisions regarding CRC screening [51].

The limitations of this review are as follows: (1) We conducted a literature search for potential studies using the specified databases (Method); (2) we considered the publication bias to be low in studies measuring prevalence, incidence, and mortality, so we did not assess for bias; and (3) studies that focused on specific Asian ethnic groups were limited. We suggest that more studies considering heterogeneous groups be carried out as the Asian American population continues to grow and diversify, especially underrepresented groups such as native Hawaiians or Pacific Islanders, South Asians, and Southeast Asians.

## Conclusions

The review collectively illustrates a concerning increase in CRC incidence among various Asian American ethnic groups, including Chinese and Korean Americans. These disparities are likely attributable to a combination of heterogeneous factors, including inadequate screening rates, insufficient educational outreach regarding CRC, and prevalent cultural barriers. It is essential to pursue further research through longitudinal cohort studies to elucidate these underlying mechanisms. Consequently, this review advocates for a more detailed categorization of the API ethnic populations, moving beyond the monolithic classification of "Asian." Furthermore, it emphasizes the need to implement preventative CRC screening initiatives within API communities, which currently exhibit lower screening rates compared to other demographic groups.

## Appendices

Full search strategy

(“Asian Americans”[MH] OR Asian American*[text word] OR AAPI[text word] OR Pacific Islander*[text word] OR Japanese American*[text word] OR Chinese American*[text word] OR Vietnamese American*[text word] OR Asian Indian American*[text word] OR Indian American*[text word] OR Cambodian American*[text word] OR Hmong American*[text word] OR Korean American*[text word] OR Filipino American*[text word] OR South Asian*[text word] OR Indonesian American*[text word] OR Pakistani American*[text word] OR Malaysian American*[text word] OR Bangladeshi American*[text word] OR Nepalese American*[text word] OR Laotian American*[Text word] OR Sri Lankan American*[text word] OR Bhutanese American*[Text word] OR Burmese American*[Text word] OR East Asian*[text word] OR Taiwanese American*[Text word] OR Mongolian American*[Text word] OR Tibetan American*[Text word] OR Thai American*[Text word] OR Macanese American*[Text word] OR Singaporean American*[Text word])

AND (social determinants of health[text word] OR “Social Determinants of Health”[MH] OR “Healthcare Disparities”[MH] OR healthcare disparities[text word] OR healthcare disparity[text word] OR socioeconomic status[text word] OR socioeconomic factors[text word] OR “Socioeconomic Factors”[MH] OR neighborhood SES[text word] OR Poverty[text word] OR Social class[text word] OR Income[text word] OR Insurance[text word] OR Occupation[text word] OR Geographic location[text word] OR Literacy[text word] OR Inequality[text word] OR Education*[text word] OR employment[text word] OR Home environment[text word] OR "Geographic Locations"[Mesh] OR ethnic enclave*[text word])

AND (“Colorectal Neoplasm”[MH] OR colorectal cancer*[text word] OR Colorectal Neoplasm*[Text word] OR colon cancer[text word] OR rectal cancer[text word])

AND (clinical outcomes[text word] OR treatment outcomes[text word] OR “Treatment Outcome”[Mesh] OR Incidence*[Text word] OR Mortality[Text word] OR Morbidity[Text word] OR "Vital Statistics"[Mesh] OR Progression free survival[Text word] OR Prognosis[Text word] OR "Prognosis"[Mesh] OR "Intraoperative Complications"[Mesh] OR Intraoperative Complication*[text word] OR surgical complication*[text word] OR "Patient Outcome Assessment"[Mesh] OR outcome*[text word] OR critical care outcome*[text word] OR "Patient Care"[Mesh] OR Patient Care[text word] OR Length of stay[Text word] OR Discharge[Text word] OR Hospitalization[Text word] OR Long term care[Text word] OR Palliative care[text word] OR terminal care[text word] OR Hospice care[text word] OR Rehabilitation[text word] OR "Rehabilitation"[Mesh] OR Activities of daily living[text word])

## References

[1] Siegel RL, Wagle NS, Cercek A, Smith RA, Jemal A (2023). Colorectal cancer statistics, 2023. CA Cancer J Clin.

[2] Miller KD, Nogueira L, Devasia T (2022). Cancer treatment and survivorship statistics, 2022. CA Cancer J Clin.

[3] Islami F, Goding Sauer A, Miller KD (2018). Proportion and number of cancer cases and deaths attributable to potentially modifiable risk factors in the United States. CA Cancer J Clin.

[4] Lin JS, Perdue LA, Henrikson NB, Bean SI, Blasi PR (2021). Screening for colorectal cancer. Updated evidence report and systematic review for the US Preventive Services Task Force. JAMA.

[5] Gu M, Thapa S (2020). Colorectal cancer in the United States and a review of its heterogeneity among Asian American subgroups. Asia Pac J Clin Oncol.

[6] Monte L, Shin H. 20.6 Million (2023). 20.6 million people in the U.S. identify as Asian, Native Hawaiian or Pacific Islander. https://www.census.gov/library/stories/2022/05/aanhpi-population-diverse-geographically-dispersed.html#:~:text=originated%20in%20Asia.-,Race,in%20combination%20with%20another%20race.

[7] Budiman A, Ruiz N (2023). Asian Americans are the fastest-growing racial or ethnic group in the U.S. https://www.pewresearch.org/short-reads/2021/04/09/asian-americans-are-the-fastest-growing-racial-or-ethnic-group-in-the-u-s/.

[8] Yin D, Morris C, Allen M, Cress R, Bates J, Liu L (2010). Does socioeconomic disparity in cancer incidence vary across racial/ethnic groups?. Cancer Causes Control.

[9] Lee RJ, Madan RA, Kim J, Posadas EM, Yu EY (2021). Disparities in cancer care and the Asian American population. Oncologist.

[10] Mulhern KC, Wahl TS, Goss LE (2017). Reduced disparities and improved surgical outcomes for Asian Americans with colorectal cancer. J Surg Res.

[11] Pan HY, Walker GV, Grant SR (2017). Insurance status and racial disparities in cancer-specific mortality in the United States: a population-based analysis. Cancer Epidemiol Biomarkers Prev.

[12] Hashiguchi Y, Hase K, Ueno H (2012). Impact of race/ethnicity on prognosis in patients who underwent surgery for colon cancer: analysis for white, African, and East Asian Americans. Ann Surg Oncol.

[13] Alshareef SH, Alsobaie NA, Aldeheshi SA, Alturki ST, Zevallos JC, Barengo NC (2019). Association between race and cancer-related mortality among patients with colorectal cancer in the United States: a retrospective cohort study. Int J Environ Res Public Health.

[14] Oh DL, Santiago-Rodríguez EJ, Canchola AJ, Ellis L, Tao L, Gomez SL (2020). Changes in colorectal cancer 5-year survival disparities in California, 1997-2014. Cancer Epidemiol Biomarkers Prev.

[15] Thompson CA, Gomez SL, Hastings KG (2016). The burden of cancer in Asian Americans: a report of national mortality trends by Asian ethnicity. Cancer Epidemiol Biomarkers Prev.

[16] Medina HN, Callahan KE, Morris CR, Thompson CA, Siweya A, Pinheiro PS (2021). Cancer mortality disparities among Asian American and native Hawaiian/Pacific Islander populations in California. Cancer Epidemiol Biomarkers Prev.

[17] Chien C, Morimoto LM, Tom J, Li CI (2005). Differences in colorectal carcinoma stage and survival by race and ethnicity. Cancer.

[18] Singh GK, Jemal A (2017). Socioeconomic and racial/ethnic disparities in cancer mortality, incidence, and survival in the United States, 1950-2014: over six decades of changing patterns and widening inequalities. J Environ Public Health.

[19] Page MJ, McKenzie JE, Bossuyt PM (2021). The PRISMA 2020 statement: an updated guideline for reporting systematic reviews. BMJ.

[20] (2023). Covidence systematic review software. http://www.covidence.org.

[21] Higgins JP, Morgan RL, Rooney AA (2024). A tool to assess risk of bias in non-randomized follow-up studies of exposure effects (ROBINS-E). Environ Int.

[22] McGuinness LA, Higgins JP (2021). Risk-of-bias VISualization (robvis): an R package and Shiny web app for visualizing risk-of-bias assessments. Res Synth Methods.

[23] Alnasser M, Schneider EB, Gearhart SL, Wick EC, Fang SH, Haider AH, Efron JE (2014). National disparities in laparoscopic colorectal procedures for colon cancer. Surg Endosc.

[24] Chu KC, Miller BA, Springfield SA (2007). Measures of racial/ethnic health disparities in cancer mortality rates and the influence of socioeconomic status. J Natl Med Assoc.

[25] Coughlin SS, Richards TB, Thompson T, Miller BA, VanEenwyk J, Goodman MT, Sherman RL (2006). Rural/nonrural differences in colorectal cancer incidence in the United States, 1998-2001. Cancer.

[26] Ellis L, Canchola AJ, Spiegel D, Ladabaum U, Haile R, Gomez SL (2018). Racial and ethnic disparities in cancer survival: the contribution of tumor, sociodemographic, institutional, and neighborhood characteristics. J Clin Oncol.

[27] Enewold L, Horner MJ, Shriver CD, Zhu K (2014). Socioeconomic disparities in colorectal cancer mortality in the United States, 1990-2007. J Community Health.

[28] Giddings BH, Kwong SL, Parikh-Patel A, Bates JH, Snipes KP (2012). Going against the tide: increasing incidence of colorectal cancer among Koreans, Filipinos, and South Asians in California, 1988-2007. Cancer Causes Control.

[29] Kcomt L, Gorey KM (2020). Chinese enclave protections among married Chinese American women: exploratory secondary analysis of colon cancer survival. Ethn Health.

[30] Kish JK, Yu M, Percy-Laurry A, Altekruse SF (2014). Racial and ethnic disparities in cancer survival by neighborhood socioeconomic status in Surveillance, Epidemiology, and End Results (SEER) Registries. J Natl Cancer Inst Monogr.

[31] Krieger N, Quesenberry C Jr, Peng T (1999). Social class, race/ethnicity, and incidence of breast, cervix, colon, lung, and prostate cancer among Asian, Black, Hispanic, and White residents of the San Francisco Bay Area, 1988-92 (United States). Cancer Causes Control.

[32] Ladabaum U, Clarke CA, Press DJ, Mannalithara A, Myer PA, Cheng I, Gomez SL (2014). Colorectal cancer incidence in Asian populations in California: effect of nativity and neighborhood-level factors. Am J Gastroenterol.

[33] Lewis-Thames MW, Langston ME, Khan S, Han Y, Fuzzell L, Xu S, Moore JX (2022). Racial and ethnic differences in rural-urban trends in 5-year survival of patients with lung, prostate, breast, and colorectal cancers: 1975-2011 Surveillance, Epidemiology, and End Results (SEER). JAMA Netw Open.

[34] Pulte D, Jansen L, Brenner H (2017). Social disparities in survival after diagnosis with colorectal cancer: contribution of race and insurance status. Cancer Epidemiol.

[35] Steinbrecher A, Fish K, Clarke CA, West DW, Gomez SL, Cheng I (2012). Examining the association between socioeconomic status and invasive colorectal cancer incidence and mortality in California. Cancer Epidemiol Biomarkers Prev.

[36] Jandova J, Ohlson E, Torres B S MR, DiGiovanni R, Pandit V, Elquza E, Nfonsam V (2016). Racial disparities and socioeconomic status in the incidence of colorectal cancer in Arizona. Am J Surg.

[37] Torre LA, Sauer AM, Chen MS Jr, Kagawa-Singer M, Jemal A, Siegel RL (2016). Cancer statistics for Asian Americans, Native Hawaiians, and Pacific Islanders, 2016: converging incidence in males and females. CA Cancer J Clin.

[38] Parker SL, Davis KJ, Wingo PA, Ries LA, Heath CW Jr (1998). Cancer statistics by race and ethnicity. CA Cancer J Clin.

[39] Gomez SL, Noone AM, Lichtensztajn DY (2013). Cancer incidence trends among Asian American populations in the United States, 1990-2008. J Natl Cancer Inst.

[40] Miller BA, Chu KC, Hankey BF, Ries LA (2008). Cancer incidence and mortality patterns among specific Asian and Pacific Islander populations in the U.S. Cancer Causes Control.

[41] Hata M, Sakamoto K, Doneza J (2010). Improvement of long-term survival of colorectal cancer in Japanese-Americans of Hawaii from 1990 to 2001. Int J Clin Oncol.

[42] Choe JH, Koepsell TD, Heagerty PJ, Taylor VM (2005). Colorectal cancer among Asians and Pacific Islanders in the U.S.: survival disadvantage for the foreign-born. Cancer Detect Prev.

[43] Redaniel MT, Laudico A, Mirasol-Lumague MR, Gondos A, Pulte D, Mapua C, Brenner H (2009). Cancer survival discrepancies in developed and developing countries: comparisons between the Philippines and the United States. Br J Cancer.

[44] Domingo JB, Chen JJ, Braun KL (2018). Colorectal cancer screening compliance among Asian and Pacific Islander Americans. J Immigr Minor Health.

[45] Zauber AG, Winawer SJ, O'Brien MJ (2012). Colonoscopic polypectomy and long-term prevention of colorectal-cancer deaths. N Engl J Med.

[46] Coughlin SS (2020). Social determinants of colorectal cancer risk, stage, and survival: a systematic review. Int J Colorectal Dis.

[47] Aizer AA, Chen MH, McCarthy EP (2013). Marital status and survival in patients with cancer. J Clin Oncol.

[48] Kim J, Kang SJ, Jo N, Kim SJ, Jang S (2025). Cancer prognosis using base excision repair genes. Mol Cells.

[49] Badial K, Lacayo P, Murakami S (2024). Biology of healthy aging: biological hallmarks of stress resistance related and unrelated to longevity in humans. Int J Mol Sci.

[50] Hwang H (2013). Colorectal cancer screening among Asian Americans. Asian Pac J Cancer Prev.

[51] Jin SW, Yoon YJ (2020). Barriers and facilitators to colorectal cancer screening among older Korean Americans: a focus group study. Soc Work Health Care.

[52] Jun J, Nan X (2018). Determinants of cancer screening disparities among Asian Americans: a systematic review of public health surveys. J Cancer Educ.

[53] Juon HS, Guo J, Kim J, Lee S (2018). Predictors of colorectal cancer knowledge and screening among Asian Americans aged 50-75 years old. J Racial Ethn Health Disparities.

[54] Kim SB (2018). Unraveling the determinants to colorectal cancer screening among Asian Americans: a systematic literature review. J Racial Ethn Health Disparities.

[55] Oh KM, Jacobsen KH (2014). Colorectal cancer screening among Korean Americans: a systematic review. J Community Health.

